# Risk factors for postoperative medical morbidity and 3-month mortality in elderly patients with hip fracture following hip arthroplasty during COVID-19 pandemic

**DOI:** 10.1186/s13018-023-03511-3

**Published:** 2023-01-22

**Authors:** Huarui Shen, Rui He, Peng Zhang, Yue He, Yingqi Liu, Guoyou Wang, Ting Li

**Affiliations:** 1 Department of Joint Surgery, The Affiliated Traditional Chinese Medicine Hospital of Southwest Medical University, Luzhou, 646000 China; 2 Department of Orthopedics, The Second People’s Hospital of Chengdu, Chengdu, 610021 People’s Republic of China; 3 Department of Joint Surgery, Sichuan Province Orthopedic Hospital, Chengdu, 610045 People’s Republic of China; 4 Sichuan Provincial Ba-Yi Rehabilitation Center (Si Chuan Provincial Rehabilitation Hospital), Chengdu City, 631000 Sichuan Province People’s Republic of China; 5 Department of Spine Surgery, Chongqing Orthopedics Hospital of Traditional Chinese Medical, Chongqing, 400000 People’s Republic of China; 6 School of Pharmacy, Southwest Medical University, Luzhou, 646000 People’s Republic of China

**Keywords:** Hip arthroplasty, COVID-19, Morality, Complications, Elderly patients

## Abstract

**Background:**

The purpose of the current study was to investigate the incidence of postoperative medical complications and 3-month mortality in patients  ≥ 70 years old with hip fracture following hip arthroplasty (HA) and independent risk factors associated with postoperative medical complications and 3-month mortality during the Coronavirus Disease 2019 (COVID-19) pandemic.

**Methods:**

A multicenter retrospective study was conducted, patients  ≥ 70 years old with HA for hip fracture under general anesthesia were included during COVID-19 and before COVID-19 pandemic. The outcome was defined as postoperative medical complications and 3-month mortality. The baseline characteristics and risk factors were collected, multivariable logistic regression was used to identify independent risk factors for postoperative medical complications and 3-month mortality.

**Results:**

A total of 1096 patients were included during COVID-19 pandemic and 1149 were included before COVID-19 pandemic in the study. Patients  ≥ 70 years with hip fracture for HA had longer fracture to operation duration (7.10 ± 3.52 vs. 5.31 ± 1.29, *P* < 0.001), and the incidence of postoperative medical complications (21.90% vs. 12.53%, *P* < 0.001) and 3-month mortality (5.20% vs. 3.22%, *P* = 0.025) was higher during COVID-2019 pandemic. Multivariate logistic regression analysis showed that dementia (OR 2.73; 95% CI 1.37–5.44; *P* = 0.004), chronic obstructive pulmonary disease (COPD) (OR 3.00; 95% CI 1.92–4.71; *P* < 0.001), longer fracture to operation duration (OR 1.24; 95% CI 1.19–1.30; *P* < 0.001) were associated with increased risk for postoperative medical complications. COPD (OR 2.10; 95% CI 1.05–4.17; *P* = 0.035), dementia (OR 3.00; 95% CI 1.11–7.94; *P* = 0.031), postoperative medical complications (OR 4.99; 95% CI 2.68–9.28; *P* < 0.001), longer fracture to operation duration (OR 1.11; 95% CI 1.04–1.19; *P* = 0.002) were associated with increased risk for 3-month mortality.

**Conclusions:**

In conclusion, we found that postoperative medical morbidity and 3-month mortality in patients with hip fracture underwent HA were 21.90% and 5.20%, respectively, during the COVID-19. COPD, dementia and longer fracture to operation duration were associated with negative outcome in patients with hip fracture underwent HA during the COVID-19.

## Background

Hip fracture is the common injuries among the elderly population in China [1, 2]. Patients with hip fractures are usually elderly, and have limited physical reserves, most patients have multiple medical comorbidities, which may increase the risk of postoperative complications and mortality [3–6]. Despite advances in multidisciplinary care, implants, anesthesia, antibiotic prophylaxis, and rehabilitation in recent years, the mortality after hip fracture surgery remains high [7, 8].

Although Sichuan and Chongqing were not at the center area of the COVID-19 pandemic, with the spread of COVID-19, many preventative measures have been implemented in all hospitals; the hospitals rapidly expanded the respiratory isolation unit and minimized the number of patients entering the ward [9, 10]. Non-emergency diagnostic imaging was postponed, and non-emergency elective surgery was postponed [11, 12]. On the other hand, fear of being infected with COVID-19 was also a reason for delayed admission.

Many studies showed that early surgery, appropriate orthopedic care, and early postoperative rehabilitation can improve the outcome among hip fracture patients [13–17], prolonged time to surgery has been associated with higher mortality and in-hospital complications in hip fracture [18, 19]. The COVID-19 pandemic has brought about major changes in the treatment of hip fracture, prolongation of time from fracture to operation, increased preoperative evaluation, and reduced postoperative rehabilitation can affect the prognosis of patients.

Due to the developing nature of the COVID-19 pandemic, the morbidity and mortality in patients hip fracture after hip arthroplasty (HA) were unclear during the COVID-19 pandemic. Therefore, in this study, we retrospectively collected data in four hospitals to investigate the effect of COVID-19 pandemic on short-term outcomes of patients  ≥ 70 years with hip fracture for HA and the incidence of postoperative medical complications and 3-month mortality and identified some independent risk factors associated with postoperative medical complications and 3-month mortality during the COVID-19 pandemic.

## Methods

### Patients and clinical data

We conducted a multicenter retrospective study. The study was carried out at four hospitals located in Sichuan and Chongqing: The Affiliated Traditional Chinese Medicine Hospital of Southwest Medical University, The Second People’s Hospital of Chengdu, Sichuan Province Orthopedic Hospital, Chongqing Orthopedics Hospital of Traditional Chinese Medical**.** Chongqing Orthopedics Hospital of Traditional Chinese Medical was more than 500-bed grade IIA hospital. The Affiliated Traditional Chinese Medical Hospital of Southwest Medical University, Sichuan Province Orthopedic Hospital, and the Second People’s Hospital of Chengdu were more than 1000-bed grade III A hospital. All patients who underwent primary HA for hip fractures were included in our study during COVID-19 pandemic (between 1 January 2020 and 30 December 2020) and before COVID-19 pandemic (between 1 January 2019 and 30 December 2019). The study was approved by the ethics committee of all the study center.

Hip fracture (femoral neck fracture or intertrochanteric fracture) was captured in the registry by using International Classification of Diseases Tenth revision (ICD-10) code reading (S72.0 and S72.1), and then, all the patients were screened manually. We included patients  ≥ 70 years old if they experienced HA for hip fracture under general anesthesia. The exclusion criteria were the following: (1) receiving bilateral arthroplasty; (2) undergoing general or regional anesthesia within 3 months; (3) revision arthroplasty; (4) multiple injuries; (5) pathological fracture or ongoing chemotherapy for a tumorous condition; (6) < 70 years old; (7) COVID-19 infection. Four clinicians (RH, HS, PZ, and YL) collected information by reviewing the Electronic Medical Record (EMR) system from four hospitals.

A data extraction form was designed and used to record the patient characteristics. The data were extracted from the Electronic Medical Record (EMR) system which include demographic characteristics [included age, gender, body mass index (BMI)], [medical history, current smoking, hypertension, hypertension, diabetes, hyperlipidemia, dementia, heart disease, chronic renal disease, chronic liver disease, chronic obstructive pulmonary disease (COPD)], and preoperative factors [albumin (ALB) and hemoglobin (HGB)].

In-hospital medical complications were defined as follows: infections (pneumonia, urinary tract infection, or other infections), vascular complications (e.g., deep venous thrombosis, pulmonary embolism, myocardial infarction, stroke), and neurologic complications (e.g., sciatic palsy).

The outcome was defined as postoperative medical complications and 3-month mortality in patient with hip fracture following HA. 3-month mortality was obtained by telephone consultation or medical records.

### Statistical analysis

The data are presented as numbers (%) or means (± standard deviations). Pearson *χ*^2^ tests were used for categorical variables. Student’s *t* tests were used to compare normally distributed variables. Mann–Whitney *U* tests were used to compare nonnormally distributed variables. Variables associated with postoperative medical complications and 3-month mortality in the univariate analyses with a *P* value < 0.20 were included in the multivariate analysis. Multivariate logistic regression analysis was performed to identify determinants independently associated with postoperative medical complications and 3-mortality. The results were expressed as the adjusted odds ratio (aOR) with their corresponding 95% confidence interval (CI). The data were analyzed using SPSS 22 software. *P* < 0.05 was considered statistically significant.

## Results

### Demographic characteristics

During the January 1, 2020 to December 30, 2020 period, a total of 1196 patients were enrolled at the four centers. Among them, 27 patients underwent hip repair, 10 patients received bilateral arthroplasty, and 53 patients with 1 or more of the other exclusion criteria were excluded, 10 patients missed information. The remaining 1096 patients were recruited in this study, there were 556 (50.73%) female and 540 (49.27%) male patients with the mean age of 76.14 ± 6.32 years (range: 70–97 years); of them, 103 (9.40%) had COPD, 352 (32.12%) patients had a history of hypertension, 41 (3.72%) patients had dementia, 301 (27.46%) had diabetes, and 533 (48.63%) had hyperlipidemia. The time from hip fracture to surgery was 7.10 ± 3.52 days.

During the January 1, 2019 to December 30, 2019 period, a total of 1264 patients were enrolled at the four centers. Among them, 30 patients underwent hip repair, 12 patients received bilateral arthroplasty, and 60 patients with 1 or more of the other exclusion criteria were excluded, 13 patients missed information. The remaining 1149 patients were recruited in this study, there were 567 (49.35%) female and 582 (50.65%) male patients with the mean age of 76.35 ± 6.40 years (range: 70–97 years); of them, 115 (10.01%) had COPD, 373 (32.46%) patients had a history of hypertension, 38 (3.31%) patients had dementia, 317 (27.59%) had diabetes, 588 (51.17%) had hyperlipidemia. The time from hip fracture to surgery was 5.31 ± 1.29 days.

Compared with patients in no COVID-19 pandemic group, patients in COVID-19 pandemic group had significantly longer fracture to operation duration (7.10 ± 3.52 vs. 5.31 ± 1.29, *P* < 0.001), the incidence of medical complications and 3-month mortality was higher in COVID-19 pandemic group (*P* < 0.005). There was no significant difference in gender, age, dementia, COPD, hypertension, current smoking, diabetes, hyperlipidemia, heart disease, chronic renal disease, chronic liver disease, ALB, RBC, operating times and the mean length of stay (LOS) (*P* > 0.05). Baseline characteristics of patients in the no COVID-19 pandemic group and COVID-19 pandemic group are shown in Table 1.

**Table 1:** Comparison of baseline characteristics between patients with no COVID-19 period and COVID-19 pandemic groups

	no COVID-19 pandemic (1149)	COVID-2019 pandemic (1096)	OR (95% CI)	P*
Age, y (mean SD)	76.35 ± 6.40	76.14 ± 6.32		0.990
Female, *n* (%)	567 (49.34)	556 (50.72)	1.06 (0.90–1.25)	0.513
BMI ≥ 24 kg/m, *n* (%)	369 (32.11)	330 (30.11)	0.91 (0.76–1.09)	0.305
Current smoking, *n* (%)	299 (26.02)	298 (27.19)	1.06 (0.88–1.28)	0.532
Hypertension, *n* (%)	373 (32.46)	352 (32.12)	0.98 (0.83–1.78)	0.861
Diabetes, *n* (%)	317 (27.59)	301 (27.46)	0.99 (0.83–1.20)	0.947
Hyperlipidemia, *n* (%)	588 (51.17)	533 (48.63)	0.903 (0.77–1.07)	0.228
Dementia, *n* (%)	38 (3.31)	41 (3.74)	1.14 (0.73–1.78)	0.577
Heart disease, *n* (%)	111 (9.66)	105 (9.58)	0.95 (0.63–1.42)	0.949
Chronic renal disease, *n* (%)	73 (6.35)	88 (8.03)	1.29 (0.93–1.78)	0.124
Chronic liver disease, *n* (%)	49 (4.26)	54 (4.93)	1.16 (0.78–1.73)	0.453
COPD, *n* (%)	115 (10.01)	103 (9.40)	0.93 (0.71–1.23)	0.625
Fracture to operation duration, d (mean SD)	5.31 ± 1.29	7.10 ± 3.52		** < 0.001**
ALB, g/l (mean SD)	33.86 ± 4.96	34.01 ± 4.89		0.947
RBC, g/l (mean SD)	135.53 ± 25.49	135.43 ± 26.07		0.996
Operating times, min (mean SD)	80.77 ± 14.28	79.55 ± 15.14		0.879
Medical complication, *n* (%)	144 (12.53)	240 (21.90)	1.96 (1.56–2.45)	** < 0.001**
3-month dead, *n* (%)	37 (3.22)	57 (5.20)	1.62 (1.06–2.47)	**0.025**

Bold indicates *P* values less than 0.05

*Comparison between no COVID-2019 pandemic and COVID-2019 pandemic groups. The data are presented as numbers (%) or mean values (± standard deviation). Pearson χ^2^ tests were used for categorical variables. Student’s *t* tests were used to compare normally distributed variables. Mann–Whitney *U* tests were used to compare nonnormally distributed variables

In COVID-19 pandemic group, 240 (21.90%) patients developed postoperative medical complications after HA. A total of 162 (14.78%) patients had infection during the hospital stay, 25 (2.28%) patients had stroke, 23 (2.10%) had deep venous thrombosis, 12 (1.09%) had myocardial infarction, 8 (0.72%) had acute kidney injury, and 10 (0.91%) had gastrointestinal bleeding. The mean LOS was 13.72 days (13.72 ± 7.51 days).

In no COVID-19 pandemic group, 144 (12.53%) patients developed postoperative medical complications after HA. A total of 104 (9.05%) patients had infection during the hospital stay, 15(1.31%) patients had stroke, 12 (1.04%) had deep venous thrombosis, 5 (0.44%) had myocardial infarction, 3 (0.26%) had acute kidney injury, and 5 (0.44%) had gastrointestinal bleeding. The mean LOS was 13.23 days (13.23 ± 6.45 days). Compared with patients with medical complications in no COVID-19 pandemic group, patients with medical complications in COVID-19 pandemic group had longer mean LOS (17.75 ± 9.00 vs. 15.23 ± 6.21, *P* = 0.009).

### Univariable models for predictors of postoperative medical complications in COVID-19 pandemic group

Univariate analysis found that fracture to operation duration, dementia and COPD were all significantly associated with increased risk for postoperative medical complications (Table 2). Compared with patients with no medical complications, patients with medical complications had significantly longer fracture to operation duration (9.30 ± 4.41 vs. 6.60 ± 2.97, *P* < 0.001), the percentage of dementia [16 (6.67%) vs. 25 (2.92%), *P* = 0.007; OR 2.37; 95% CI 1.25–4.52; *P* = 0.007] and COPD [47 (19.17%) vs. 57 (6.66%), *P* < 0.001; OR 3.32; 95% CI 1.19–5.05; *P* < 0.001]. The mean LOS was 17.75 ± 9.00 days for medical complications and 12.59 ± 6.62 days for no medical complications, respectively, the difference between the two groups was about 5 days (*P* < 0.001).

**Table 2:** Comparison of baseline characteristics between patients with no complications and complications groups

	No complications group (856)	Complications group (240)	OR (95% CI)	P*
Age, y (mean SD)	76.18 ± 6.39	76.01 ± 6.10		0.588
Female, *n* (%)	438 (51.17)	118 (49.17)	0.92 (0.69–1.23)	0.584
BMI ≥ 24 kg/m, *n* (%)	264 (30.84)	66 (27.50)	0.85 (0.62–1.17)	0.319
Current smoking, *n* (%)	241 (28.15)	57 (23.75)	0.80 (0.57–1.11)	0.175
Hypertension, *n* (%)	272 (31.78)	80 (33.33)	1.07 (0.79–1.46)	0.648
Diabetes, *n* (%)	239 (27.92)	62 (25.83)	0.90 (0.65–1.25)	0.522
Hyperlipidemia, *n* (%)	414 (43.86)	119 (49.58)	1.05 (0.79–1.40)	0.738
Dementia, *n* (%)	25 (2.92)	16 (6.67)	2.37 (1.25–4.52)	**0.007**
Heart disease, *n* (%)	81 (9.46)	24 (10.00)	1.06 (0.66–1.72)	0.803
Chronic renal disease, *n* (%)	69 (8.06)	19 (7.92)	0.89 (0.48–1.62)	0.942
Chronic liver disease, *n* (%)	40 (4.67)	14 (5.83)	1.26 (0.68–2.36)	0.463
COPD, *n* (%)	57 (6.66)	46 (19.17)	3.32 (1.19–5.05)	** < 0.001**
Fracture to operation duration, d (mean SD)	6.60 ± 2.97	9.30 ± 4.41		** < 0.001**
ALB, g/l (mean SD)	34.06 ± 4.99	33.84 ± 4.53		0.465
RBC, g/l (mean SD)	135.06 ± 25.98	136.77 ± 26.34		0.362
Operating times, min (mean SD)	80.57 ± 14.29	79.77 ± 14.86		0.420

Bold indicates *P* values less than 0.05

*Comparison between no complications and complications groups. The data are presented as numbers (%) or mean values (± standard deviation). Pearson *χ*^2^ tests were used for categorical variables. Student’s *t* tests were used to compare normally distributed variables. Mann–Whitney *U* tests were used to compare nonnormally distributed variables

### Multivariable models on the association between risk factors and postoperative medical complications in COVID-19 pandemic group

Unadjusted logistic regression analysis identified longer fracture to operation duration (OR 1.24; 95% CI 1.19–1.29; *P* < 0.001), higher percentage of dementia (OR 2.37; 95% CI 1.25–4.52; *P* = 0.009) and COPD (OR 3.32; 95% CI 2.19–5.05; *P* < 0.001) as risk factors associated with postoperative medical complications. When variables associated with postoperative medical complications in the univariate analyses with a *P* value < 0.20 were included in the multivariate analysis (adjusted for current smoking, dementia, COPD, fracture to operation duration), the results showed that dementia (OR 2.73; 95% CI 1.37–5.44; *P* = 0.004), COPD(OR 3.00; 95% CI 1.92–4.71; *P* < 0.001), longer fracture to operation duration (OR 1.24; 95% CI 1.19–1.30; *P* < 0.001) were associated with increased risk for postoperative medical complications (Table 3).

**Table 3:** Multivariable models showing factors associated with postoperative medical complications

	OR (95% CI)	P*
Dementia	2.73 (1.37–5.44)	**0.004**
COPD	3.00 (1.92–4.71)	** < 0.001**
Fracture to operation duration	1.24 (1.19–1.30)	** < 0.001**

Bold indicates *P* values less than 0.05

*Multivariable adjusted for current smoking, dementia, COPD, fracture to operation duration

### Univariable Models for Predictors of death in COVID-19 pandemic group

Fifty-seven out of 1096 (5.20%) patients had died at 3 months. Baseline characteristics of patients in the survivor and dead groups were compared (Table 4). At baseline, dead group showed significantly longer fracture to operation duration (9.80 ± 4.58 vs. 7.05 ± 3.40, *P* < 0.001), the percentage of COPD [14 (24.56%) vs. 89 (8.57%); OR 3.48; 95% CI 1.83–6.60; *P* < 0.001] and postoperative medical complications [37 (64.91%) vs203 (19.54%); OR 8.03; 95% CI 4.52–14.26; *P* < 0.001] than patients with survivor group (Table 4).

**Table 4:** Comparison of baseline characteristics between patients with survival and death groups

	Survival group (1039)	dead group (57)	OR (95% CI)	P*
Age, y (mean SD)	76.70 ± 6.30	77.46 ± 6.51		0.154
Female, *n* (%)	529 (50.91)	27 (47.47)	0.87 (0.51–1.48)	0.602
BMI ≥ 24 kg/m, *n* (%)	311 (29.93)	19 (33.33)	1.17 (0.66–2.06)	0.586
Current smoking, *n* (%)	284 (27.334)	14 (24.56)	0.87 (0.47–1.61)	0.647
Hypertension, *n* (%)	333 (32.05)	19 (33.33)	1.06 (0.60–1.87)	0.840
Diabetes, *n* (%)	282 (27.14)	19 (33.33)	1.34 (0.76–2.37)	0.308
Hyperlipidemia, *n* (%)	501 (48.22)	32 (56.14)	1.38 (0.80–2.35)	0.244
Dementia, *n* (%)	35 (3.37)	6 (10.53)	3.38 (1.36–8.39)	0.006
Heart disease, *n* (%)	99 (9.53)	6 (10.53)	1.12 (0.47–2.67)	0.803
Chronic renal disease, *n* (%)	85 (8.18)	3 (5.26)	0.62 (0.19–2.04)	0.430
Chronic liver disease, *n* (%)	52 (5.00)	2 (3.51)	0.69 (0.16–2.91)	0.611
COPD, *n* (%)	89 (8.57)	14 (24.56)	3.48 (1.83–6.60)	** < 0.001**
Fracture to operation duration, d (mean SD)	7.05 ± 3.40	9.80 ± 4.58		** < 0.001**
ALB, g/l (mean SD)	33.99 ± 4.92	34.49 ± 4.26		0.596
RBC, g/l (mean SD)	135.43 ± 26.11	135.43 ± 25.32		0.993
Operating times, min (mean SD)	80.47 ± 14.28	78.89 ± 16.63		0.366
Postoperative medical complications, *n* (%)	203 (19.54)	37 (64.91)	8.03 (4.52–14.26)	** < 0.001**

Bold indicates *P* values less than 0.05

^*^Comparison between survival and dead groups. The data are presented as numbers (%) or mean values (± standard deviation). Pearson *χ*^2^ tests were used for categorical variables. Student’s *t* tests were used to compare normally distributed variables. Mann–Whitney *U* tests were used to compare nonnormally distributed variables

### Multivariable Models on the Association between risk factors and 3-month mortality in COVID-19 pandemic

Unadjusted logistic regression analysis identified longer fracture to operation duration (OR 1.20; 95% CI 1.12–1.28; *P* < 0.001), the percentage of COPD (OR 3.48; 95% CI 1.83–6.60; *P* < 0.001), dementia (OR 3.38; 95% CI 1.34–8.39; *P* < 0.001) and postoperative medical complications (OR 7.62; 95% CI 4.33–13.41; *P* < 0.001) as risk factors associated with 3-mortality. When variables associated with 3-mortality in the univariate analyses with a *P* value < 0.20 were included in the multivariate analysis (adjusted for age, dementia, COPD, fracture to operation duration and postoperative medical complications), the results showed that percentage of COPD (OR 2.10; 95% CI 1.05–4.17; *P* = 0.035), dementia (OR 3.00; 95% CI 1.11–7.94; *P* = 0.031), postoperative medical complications (OR 4.99; 95% CI 2.68–9.28; *P* < 0.001), longer fracture to operation duration (OR 1.11; 95% CI 1.04–1.19; *P* = 0.002) were associated with increased risk for 3-month mortality (Table 5).

**Table 5:** Multivariable models showing factors associated with 3-month mortality

	OR (95% CI)	P*
COPD	2.10 (1.05–4.17)	**0.035**
Dementia	3.00 (1.11–7.94)	**0.031**
Fracture to operation duration	1.11 (1.04–1.19)	**0.002**
Postoperative medical complications	4.99 (2.68–9.28)	** < 0.001**

Bold indicates *P* values less than 0.05

*Multivariable adjusted for age, dementia, COPD, fracture to operation duration and postoperative medical complications

## Discussion

In this study, we reported the effect of COVID-19 pandemic on short-term outcomes of patients  ≥ 70 years with hip fracture for HA and investigated incidence of postoperative medical complications and 3-month mortality and identified some independent risk factors associated with incidence of postoperative medical complications and 3-month mortality during the COVID-19 pandemic in four hospitals. Few patients were operated within 48 h of hospital admission during the COVID-19 pandemic, as envisaged by the standardized organizational model [20, 21]. In this study, we found that patients  ≥ 70 years with hip fracture for HA had longer fracture to operation duration and the incidence of postoperative medical complications and 3-month mortality was higher during COVID-19 pandemic. Several factors associated with increased the risk for postoperative medical complications and 3-month mortality.

In this study, the results showed that the most common complications were infection, including pneumonia, urinary tract infection or other infections. Patients who experienced postoperative medical complications had longer fracture to operation duration, percentage of dementia and COPD. Multivariate logistic regression analyses indicated that dementia, COPD and longer fracture to operation duration were associated with postoperative medical complications in patients  ≥ 70 years old after HA for hip fracture, and the results also showed that COPD, longer fracture to operation duration and perioperative complications were associated with the increased risk for 3-month mortality.

Dementia is a very common medical comorbidity in elderly patients with hip fracture, it is associated with an increased risk of poor postoperative outcomes, including increased mortality and morbidity [22, 23]. The reasons may be that patients with dementia have more severe medical comorbidities and have a poorer reserve for physical function. In this study, the incidence of dementia was 3.46%, which was much lower than previous studies [22–24]. The reason might be that this study was a retrospective study, and most of the dementia history was provided by the patients' family members and patients. However, in this study the results showed that patients with dementia experienced higher complications after THA (40%, 12/30), which was consistent with previous studies [25, 26]. Previous studies also showed that preoperative risk factors for in-hospital mortality were advanced age, presence of comorbid diseases such as dementia, and renal or cerebrovascular disease [27, 28], and patients with a diagnosis of dementia had higher in-hospital mortality (*P* < 0.001) [28]. The two retrospective studies included a combined total of 40,958 and showed that patients with dementia and age over 90 years old have a higher 90 day and 1-year mortality with hazards of 88% (*P* = 0.01) and 75% (*P* = 0.01), respectively [29, 30]. In this study, dementia was not associated with 3-month mortality, which may be related to the low number of dementia patients. In our study, we found that dementia was associated with the increased the risk for postoperative medical complications, and latter was associated with the increased the risk for 3-month mortality. Therefore, postoperative care processes such as rehabilitation may provide for better surveillance of postoperative complications while they are in this setting and participation in rehabilitation may help prevent complications.

During the epidemic period, all aspects of social life were affected, the government adopted a series of containment measures to limit the spread of COVID-19, including extensive testing, quarantine of cases and prohibiting gathering [31–33]. Patients with hip fracture were more likely to reside in residential home, which may lead to longer time from fracture to operation. Delay for hip fracture surgery is undesirable. When patients sustain a hip fracture, they are forced to lie flat in a bed and are either in pain or needing analgesic medications, which often have side effects [34, 35]. Previous study found that patients who received operation within 2 days after injury had lower in-hospital mortality and had better outcomes [30, 36, 37]. In this study, we found the mean fracture to operation duration was 7.10 days in COVID-19 pandemic group, and 5.31 days in no COVID-19 pandemic group, the difference between the two groups was about 2 days, and the incidence of postoperative medical complications and 3-month mortality was 21.90% and 5.20% for COVID-19 pandemic group compared to 12.53% and 3.22% for no COVID-19 pandemic group in patients ≥ 70 years with hip fracture following HA.

The key to advocating early surgery is to restore motor function at an early stage and to reduce complications associated with bed rest. Such complications can occur not only postoperatively but also preoperatively. A previous study showed that patients admitted one week after injury had higher incidence of preoperative pneumonia and lower level of albumin [37]. Our study also showed that longer fracture to operation duration was associated with an increased risk for postoperative medical complications. We speculate the intrinsic link is that with admission delay continuing and bedridden time prolonging, preoperative pneumonia would appear and aggravate, and nutritional status would deteriorate.

COPD has been associated with impaired bone metabolism, patients undergoing THA with underlying COPD face a higher rate of comorbidities, respiratory complications, implant complications and revision surgeries, than patients without COPD [38]. Studies have shown an increased risk of myocardial infarction, pneumonia, unplanned reintubation, deep infection and 30-day readmission rates [39–44]. Patients with COPD present an additional challenge, given decreased pulmonary reserve and associated chronic diseases, making them susceptible to further complications [45, 46]. In our studies, the results showed that patients with COPD, hip fracture and prolongation of time to surgery presented additional challenges, patients undergoing HA had an increased risk of postoperative medical complications and 3-month mortality, which consists of previous studies [45, 46].

In this study, we found that there was no significant difference in mean LOS between COVID-19 pandemic and no COVID-19 pandemic groups, but mean LOS of patients with postoperative medical complications was significantly longer than those who without postoperative medical complications, and the mean LOS of patients with medical complications during the COVID-19 pandemic was also significantly longer than those who in no COVID-19 pandemic. We speculated that the possible reason for these results was that during the COVID-19 pandemic, prolonged time to surgery might cause more serious postoperative medical complications, so longer hospitalization was needed for patients.

There were some limitations of this study. First, this is a retrospective study, history of dementia was provided by patients and families, and the incidence of dementia in patients with hip fractures may be underestimated, which may have resulted in response bias. Second, Sichuan and Chongqing are not the COVID-19 pandemic central area, the results may not be representative. Third, postoperative indicators (such as plasma albumin levels, hemoglobin levels, duration spent in intensive care unit, postoperative rehabilitation and care level) may have affected the outcomes. Fourthly, the specific operative technique, family history, physical activity and dietary habits were not capture. In addition, we lacked data on APACHE scores and the approach of the surgery. Despite the limitations mentioned above, this study was a multicenter study with a large number of patients; our results provide valuable information on morbidity and mortality in elderly patients with total hip arthroplasty for hip fracture during COVID-19 pandemic.

## Conclusions

In conclusion, we found that prolongation of time to surgery was common, and postoperative medical morbidity and 3-month mortality in patients with hip fracture underwent HA were 22.29% and 5.89% during the COVID-19 pandemic, respectively, which were significantly higher than those before COVID-19 pandemic. COPD, dementia and longer fracture to operation duration were associated with negative outcome in patients with hip fracture underwent HA. In the era of COVID-19, the healthcare system must adopt new approaches (including provision of rapid COVID-19 testing, early surgery and ensuring availability of operating rooms and staff, multidisciplinary approach) to reduce mortality and medical complications in patients with hip fractures underwent HA.

## Data Availability

The datasets used and/or analyzed during the current study are available from the corresponding author on reasonable request.

## Abbreviations

CI: Confidence interval
M: Mean
OR: Odds ratio
SD: Standard deviation
COVID-19: Coronavirus disease 2019
HA: Hip arthroplasty
COPD: Chronic obstructive pulmonary disease
